# Fracture resistance of teeth obturated with different root canal sealers: a systematic review

**DOI:** 10.3389/fdmed.2026.1868502

**Published:** 2026-07-02

**Authors:** Aditya Shetty, Lakshmi Nidhi Rao, Aishwarya Nair, Junaid Ahmed, Vidya G. Doddawad, Heeresh Shetty

**Affiliations:** 1 Department of Conservative Dentistry and Endodontics, A B Shetty Memorial Institute of Dental Sciences, Nitte (Deemed to be University), Mangalore, India; 2 Department of Oral Medicine and Radiology, Manipal College of Dental Sciences Mangalore, Manipal Academy of Higher Education, Manipal, India; 3 Dept of Oral Pathology and Microbiology, JSS Dental College and Hospital, A Constituent College of JSS Academy of Higher Education and Research, Mysore, India; 4 Dept of Conservative Dentistry, Nair Hospital Dental College, Mumbai, India

**Keywords:** endodontically treated teeth, fracture resistance, mechanical strength, obturation materials, root canal sealers

## Abstract

**Background:**

Endodontically treated teeth are more prone to fracture due to structural loss during canal preparation and alterations in dentin biomechanics. Root canal sealers and obturation materials contribute not only to sealing ability but also to potential reinforcement of the remaining tooth structure. However, their influence on fracture resistance remains unclear due to variability in study designs and outcomes.

**Objective:**

To systematically evaluate and synthesize the available evidence on the fracture resistance of endodontically treated teeth obturated with different root canal sealers and materials.

**Methods:**

A comprehensive search was conducted across MEDLINE (via PubMed), Scopus, Web of Science, and the Cochrane Library, supplemented by grey literature from Google Scholar. A total of 549 records were identified, of which 24 studies met the eligibility criteria and were included in the qualitative synthesis. Among these, 22 were *in vitro* studies, one was an *in vivo* animal study, and one was an *ex vivo* study. Two independent reviewers performed study selection and data extraction. Due to methodological heterogeneity, a qualitative synthesis was performed.

**Results:**

Obturation with root canal sealers generally improved fracture resistance compared with instrumented but unobturated controls, indicating a reinforcing effect. Epoxy resin-based and calcium silicate-based sealers demonstrated comparable performance, while zinc oxide eugenol-based sealers showed limited reinforcement. Considerable heterogeneity in tooth selection, loading conditions, and testing protocols limited direct comparisons.

**Conclusion:**

Root canal sealers appear to enhance the mechanical strength of endodontically treated teeth; however, no specific sealer demonstrates clear superiority. Standardized experimental models and clinical studies are needed to determine their clinical significance.

**Systematic Review Registration:**

Unique Identifier, CRD420251163273.

## Introduction

Vertical root fracture and catastrophic structural failure remain among the most frequent causes of extraction of endodontically treated teeth, significantly affecting long-term prognosis and treatment success. Although elimination of infection and achievement of an adequate seal are primary goals of endodontic therapy, preservation of tooth structure is equally critical for maintaining biomechanical integrity. The process of endodontic treatment inherently involves removal of dentin during access cavity preparation and canal instrumentation, which reduces stiffness and alters stress distribution within the root, predisposing teeth to fracture under functional and parafunctional loading conditions (1–3).

In addition to structural loss, chemical procedures such as irrigation and intracanal medicament placement may modify dentin's organic matrix and moisture content, potentially influencing its viscoelastic properties. Sodium hypochlorite, for example, has been shown to degrade collagen, thereby reducing dentin toughness and fatigue resistance (4). Consequently, the reinforcing potential of root canal obturation materials has been widely investigated as a strategy to compensate for the weakened tooth structure.

Root canal sealers play a fundamental role in obturation by filling voids between the core material and dentinal walls, improving adaptation and preventing microleakage (5). Beyond this sealing function, contemporary materials have been proposed to enhance fracture resistance through micromechanical bonding or bioactive interactions at the sealer–dentin interface. Epoxy resin–based sealers, such as AH Plus, are believed to form a hybridized interfacial layer, allowing stress distribution along the canal walls and potentially reinforcing the root (6). Meanwhile, calcium silicate–based sealers have gained attention because of their bioactive properties, including calcium ion release and formation of hydroxyapatite-like deposits that may promote intratubular mineralization and interfacial strengthening (7).

Conversely, traditional zinc oxide eugenol sealers exhibit limited adhesion to dentin and may undergo partial dissolution over time, potentially compromising structural reinforcement (8). Glass ionomer–based sealers have also been suggested to provide chemical bonding to dentin, though their reinforcing effect remains inconsistent in the literature (9). These differences in physicochemical behavior highlight the importance of material selection when considering the long-term mechanical performance of endodontically treated teeth.

Despite extensive laboratory research, the relationship between sealer type and fracture resistance remains controversial. Many *in-vitro* studies report increased fracture resistance in obturated roots compared with instrumented but unfilled controls, supporting the concept that obturation contributes to structural reinforcement (10). However, other investigations have found minimal or no significant differences between sealer categories, suggesting that the remaining dentin thickness and overall tooth morphology may play a more dominant role than the filling material itself (11).

Interpretation of existing evidence is further complicated by substantial heterogeneity across studies. Variations in tooth type, canal preparation techniques, obturation methods, loading direction, crosshead speed, and simulation of periodontal ligament conditions can significantly influence fracture resistance outcomes (12). Moreover, most available data originate from *in-vitro* models, which cannot fully replicate intraoral biomechanical stresses, cyclic fatigue, and aging processes. As a result, extrapolation of laboratory findings to clinical performance remains limited.

Given these inconsistencies, a comprehensive and systematic evaluation of the literature is essential to clarify the current state of evidence and identify potential trends regarding the reinforcing effect of different root canal sealers. Synthesizing available data will also help highlight methodological limitations and guide future research toward standardized testing protocols and clinically relevant study designs.

Therefore, the aim of this systematic review is to critically appraise and synthesize existing evidence on the fracture resistance of endodontically treated teeth obturated with different root canal sealers, thereby providing a clearer understanding of whether material selection influences biomechanical outcomes.

## Materials and methods

### Protocol registration

The protocol for this systematic review was prospectively registered in the PROSPERO (Registration No.: CRD420251163273) to ensure transparency, methodological rigor, and to minimize the risk of selective reporting. Reporting of this review adheres to the Preferred Reporting Items for Systematic Reviews and Meta-Analyses (PRISMA) guidelines.

### Focused question

The review addressed the following question:

Do different root canal sealers influence the fracture resistance of endodontically treated teeth?

### Eligibility criteria

Studies were considered eligible if they evaluated the fracture resistance or mechanical strength of endodontically treated teeth obturated with root canal sealers. Both laboratory-based investigations (*in vitro* and *ex vivo*), animal studies, and clinical studies were included provided they compared at least one root canal sealer with another sealer type or a control group and reported quantitative fracture resistance outcomes. Only articles published in English were considered. Studies were excluded if they were case reports, narrative reviews, editorials, or conference abstracts, as well as studies that did not specifically evaluate fracture resistance, assessed only obturation techniques without identifying the sealer used, or failed to provide sufficient quantitative data for analysis.

### Search strategy

A comprehensive electronic literature search was conducted to identify relevant studies from database inception to the date of the final search. The following databases were systematically searched: MEDLINE via PubMed, Scopus, Web of Science, and the Cochrane Library. To ensure comprehensive coverage and minimize publication bias, grey literature was explored using Google Scholar. In addition, the reference lists of all included studies and relevant reviews were manually screened to identify any additional eligible articles that may have been missed during the electronic search.The search strategy combined controlled vocabulary and free-text terms related to endodontically treated teeth, root canal sealers, obturation materials, and fracture resistance. Boolean operators (AND, OR) were used to combine terms.

### Study selection

All retrieved records were imported into reference management software, and duplicates were removed. Two independent reviewers screened titles and abstracts for eligibility. Full-text articles were then assessed against the inclusion criteria. Any disagreements were resolved through discussion or consultation with a third reviewer.

### Data extraction

Data were extracted independently by two reviewers using a standardized form. The following information was recorded:

- Author and year
- Study design
- Sample size and tooth type
- Sealer type
- Obturation technique
- Testing conditions
- Fracture resistance outcomes

### Risk of bias assessment

The methodological quality of the included studies was assessed using the QUIN tool, a validated instrument specifically developed for evaluating *in vitro* dental research. The QUIN tool assesses multiple domains, including clearly stated aims, sample size justification, randomization, standardization of specimens, operator blinding, outcome assessment, and appropriateness of statistical analysis. Each domain is scored as adequately specified, inadequately specified, or not specified. Based on the cumulative score, studies were categorized as having low, moderate, or high risk of bias (Table 1). The use of the QUIN tool was justified due to its specificity and reliability in assessing methodological rigor in laboratory-based dental studies (13).

**Table 1:** Risk of bias assessment.

Study (Author, Year)	Aims	Sample Size	Randomization	Standardization	Operator blinding	Outcome blinding	Statistics	Total (/14)	%	Risk
Salim & Saleem (2025) (14)	2	0	0	2	0	0	2	7	50%	Moderate
Saba & ElAsfouri (2019) (15)	2	0	0	2	0	0	2	6	43%	High
Bhat et al. (2012) (16)	2	0	0	2	0	0	2	6	43%	High
Ersoy & Evcil (2015) (17)	2	0	0	2	0	0	2	7	50%	Moderate
Alkahtany et al. (2021) (18)	2	0	0	2	0	0	2	7	50%	Moderate
Al-Hiyasat et al. (2023) (19)	2	0	0	2	0	0	2	7	50%	Moderate
Yusufoglu et al. (2019) (20)	2	0	0	2	0	0	2	6	43%	High
Özyürek & Türker (2019) (21)	2	0	0	2	0	0	2	6	43%	High
Phukan et al. (2017) (22)	2	0	0	2	0	0	2	6	43%	High
Hassan & Hassan (2023) (23)	2	0	0	2	0	0	2	7	50%	Moderate
Almohaimede et al. (2020) (24)	2	0	0	2	0	0	2	6	43%	High
Ismail et al. (2023) (25)	2	0	0	2	0	0	2	6	43%	High
Elnawawy et al. (2023) (26)	2	0	0	2	0	0	2	6	43%	High
Dibaji et al. (2017) (27)	2	0	0	2	0	0	2	6	43%	High
Langalia et al. (2015) (28)	2	0	0	2	0	0	2	6	43%	High
Guneser et al. (2016) (29)	2	0	0	2	0	0	2	6	43%	High
Huang et al. (2023) (30)	2	0	0	2	0	0	2	6		High
El-Gamal et al. (2024) (31)	2	0	0	2	0	0	2	7	50%	Moderate
Abdallah et al. (2023) (32)	2	0	0	2	0	0	2	6	43%	High
Yendrembam et al. (2019) (33)	2	0	0	2	0	0	2	6	43%	High
Issar et al. (2021) (34)	2	0	0	2	0	0	2	6	43%	High
Hajihassani et al. (2022) (35)	2	0	0	2	0	0	2	6	43%	High
Muraleedhar et al. (2022) (36)	2	0	0	2	0	0	2	6	43%	High
Shukri & Gawish (2024) (37)	2	0	0	2	0	0	2	6	43%	High

### Data synthesis

Due to heterogeneity in study designs, tooth types, and fracture testing protocols, a quantitative meta-analysis was not performed. Instead, results were synthesized qualitatively by grouping studies according to sealer type and comparing trends in fracture resistance outcomes.

The electronic database search identified *n* **=** **511** records from MEDLINE via PubMed (*n* = 168), Scopus (*n* = 142), Web of Science (*n* = 126), and the Cochrane Library (*n* = 76). An additional *n* **=** **38** records were identified through Google Scholar and manual screening of reference lists, yielding a total of *n* **=** **550** records.

After removal of *n* **=** **123** duplicates, *n* **=** **426** unique records remained for title and abstract screening. Of these, *n* **=** **372** records were excluded as they did not meet the eligibility criteria, primarily because they did not evaluate fracture resistance or did not involve root canal sealers.

The full texts of *n* **=** **54** articles were assessed for eligibility. Of these, *n* **=** **30**were excluded for the following reasons: absence of quantitative fracture resistance data (*n* = 11), no comparison between sealer types (*n* = 9), evaluation of obturation techniques only (*n* = 5), and insufficient methodological details (*n* = 4).

**Figure 1:**
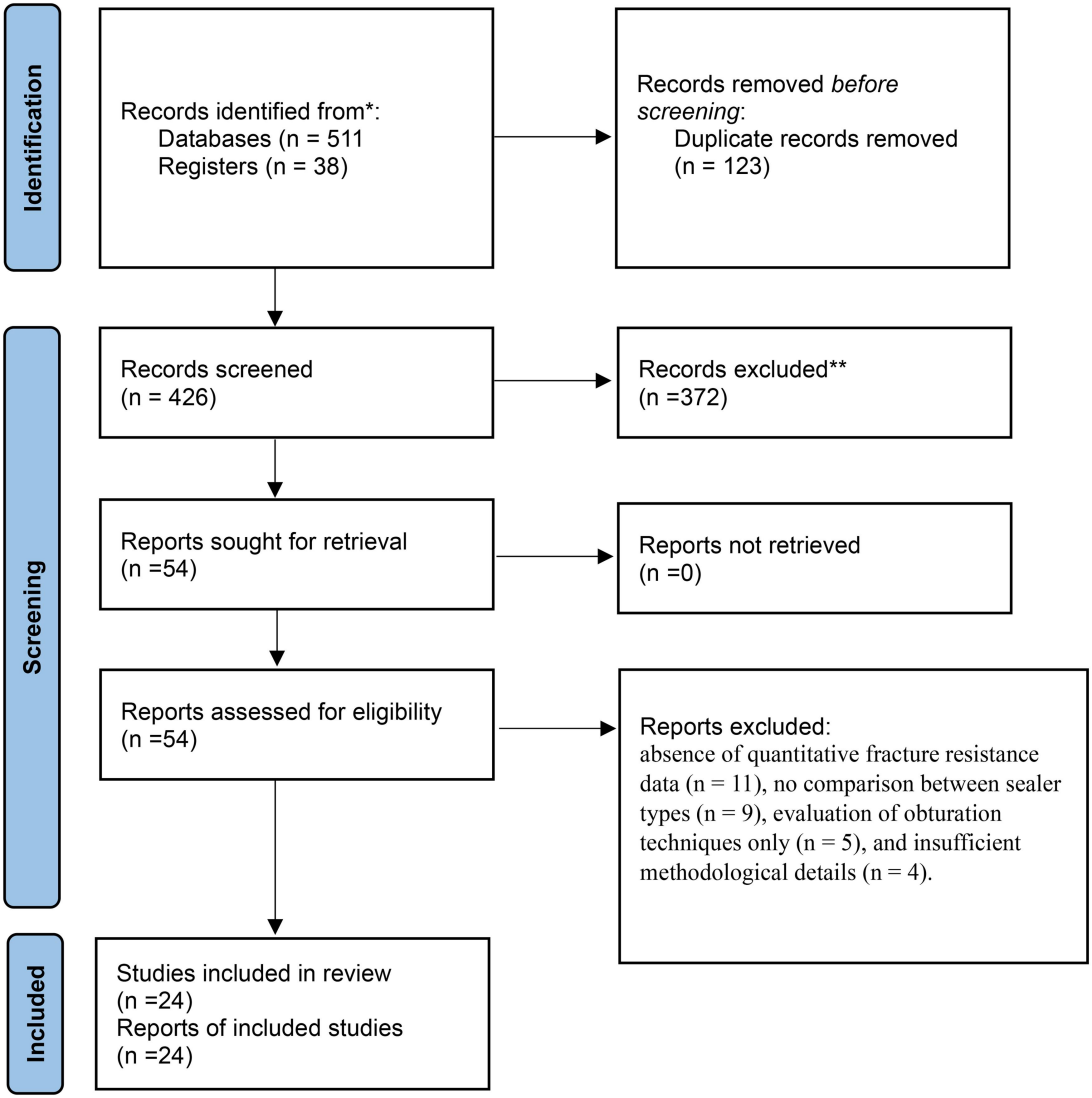
Study selection (PRISMA flow).

Finally, *n* **=** **24** studies met the inclusion criteria and were included in the qualitative synthesis (Figure 1). Owing to substantial heterogeneity in tooth types, testing conditions, and outcome reporting, a meta-analysis was not undertaken.

## Results

### Characteristics of included studies

A total of 24 studies fulfilled the predefined eligibility criteria and were included in the qualitative synthesis (Table 2). Of these, 22 were *in vitro* studies, one was an *in vivo* animal study, and one was an *ex vivo* investigation, indicating that the currently available evidence is predominantly laboratory-based.

**Table 2:** Study characteristics.

Author and year	Study design	Sample size	Sealer type	Obturation technique
Salim F, Saleem BM (2025) (14)	*In vitro* study	90	AH Plus (epoxy resin-based), MTA-based sealer, Bioceramic sealer (e.g., EndoSequence BC)	Cold lateral compaction, Single-cone technique, Warm vertical compaction
Saba AA, ElAsfouri HA (2019) (15)	*In vitro*	60	AH Plus, Endoseal MTA, BioRoot RCS	Single-cone technique
Bhat SS et al. (2012) (16)	*In vitro*	60	AH Plus, Apexit, Endoflas FS	Lateral compaction
Ersoy I, Evcil MS (2015) (17)	*In vitro*	80	AH Plus, MTA-based sealer	Lateral compaction, Warm vertical
Alkahtany MF et al. (2021) (18)	*In vitro*	120	Bioceramic, AH Plus	Single-cone, Lateral compaction, Warm vertical
Al-Hiyasat AS et al. (2023) (19)	*In vitro*	100	Resin-based, Bioceramic, MTA-based	Lateral compaction, Single-cone
Yusufoglu SI et al. (2019) (20)	*In vitro*	90	AH Plus, MTA Fillapex, BC sealer	Lateral compaction
Özyürek EU, Türker SA (2019) (21)	*In vitro*	60	AH Plus, EndoSequence BC, MTA Fillapex	Single-cone
Phukan AH et al. (2017) (22)	*In vitro*	75	AH Plus, MTA Fillapex, Apexit, ZOE	Lateral compaction
Hassan N, Hassan R (2023) (23)	*Ex vivo*	80	Bioceramic, AH Plus	Single-cone, Warm vertical
Almohaimede A et al. (2020) (24)	*In vitro*	60	Bioceramic, AH Plus	Single-cone
Ismail SM et al. (2023) (25)	*In vivo* (animal)	Dog teeth	Calcium silicate sealers, Resin sealer	Not specified
Elnawawy MS et al. (2023) (26)	*In vitro*	80	Bioceramic, Resin-based	Single-cone
Dibaji F et al. (2017) (27)	*In vitro*	60	AH Plus, MTA Fillapex, EndoSequence BC	Lateral compaction
Langalia AK et al. (2015) (28)	*In vitro*	60	Resin-based adhesive sealers	Lateral compaction
Guneser MB et al. (2016) (29)	*In vitro*	50	BioRoot RCS (calcium silicate)	Single-cone
Huang G et al. (2023) (30)	*In vitro*	72	Bioactive glass-based sealer	Single-cone
El-Gamal MK et al. (2024) (31)	*In vitro*	90	Bioceramic sealer	Lateral compaction, Warm vertical
Abdallah Y et al. (2023) (32)	*In vitro*	60	Bioceramic, Resin-based	Single-cone
Yendrembam B et al. (2019) (33)	*In vitro*	80	AH Plus, MTA Fillapex, Bioceramic	Lateral compaction
Issar R et al. (2021) (34)	*In vitro*	60	Two resin-based sealers	Lateral compaction
Issar R et al. (2021) (34)	*In vitro*	60	Two resin-based sealers	Lateral compaction
Hajihassani N et al. (2022) (35)	*In vitro*	50	Bioceramic, Resin-based	Single-cone
Muraleedhar AV et al. (2022) (36)	*In vitro*	60	AH Plus, MTA Fillapex, BioRoot RCS	Single-cone
Shukri Bm, Gawish As (2024) (37)	*In Vitro*	80 Extracted Teeth	Ah Plus, Mta Fillapex, Onefill, Neosealer	Lateral Compaction

All included studies used teeth subjected to standardized endodontic procedures before mechanical testing. Most investigations adopted a comparative design, evaluating different root canal sealers against each other or against control groups such as unfilled canals or gutta-percha without sealer. Random allocation of samples was reported in several studies; however, details regarding allocation procedures and blinding were inconsistently described.

Sample sizes ranged from 30 to 120 specimens, with most studies including 10–20 samples per group. Single-rooted permanent teeth, particularly mandibular premolars, were the most frequently used specimens, followed by maxillary incisors. Root length was commonly standardized by decoronation at the cementoenamel junction, resulting in lengths of approximately 12–15 mm. Canal preparation methods were generally comparable across studies, with apical preparation sizes typically ranging from ISO #30 to #50.

The evaluated sealers were broadly categorized as epoxy resin–based, calcium silicate–based, zinc oxide eugenol–based, and other experimental or glass ionomer–based materials. Calcium silicate and resin-based materials were the most frequently investigated categories.

Obturation approaches varied among studies and included cold lateral compaction, single-cone techniques, and warm vertical compaction.

Mechanical testing was performed using a universal testing machine in all studies, with compressive load applied until root fracture occurred. Most investigations used vertical loading along the long axis of the root, whereas a minority employed oblique loading angles. Crosshead speeds ranged from 0.5 to 1 mm/min. Specimens were commonly embedded in acrylic resin, and several studies incorporated periodontal ligament simulation materials.

The primary outcome measure was maximum load to fracture, expressed in Newtons. Some studies additionally evaluated fracture patterns, although this parameter was not consistently reported. A detailed summary of the fracture resistance outcomes associated with each sealer evaluated in the included studies is presented in Table 3.

**Table 3:** Fracture resistance outcomes.

Author and year	Best performing sealer	Comparative outcome	Statistical significance
Salim F, Saleem BM (2025) (14)	Bioceramic	>MTA > Resin	Significant
Saba AA, ElAsfouri HA (2019) (15)	BioRoot RCS	>MTA > Resin	Significant
Bhat SS et al. (2012) (16)	AH Plus	>others	Significant
Ersoy I, Evcil MS (2015) (17)	MTA + Warm vertical	Technique dependent	Significant
Alkahtany MF et al. (2021) (18)	Bioceramic	>Resin	Significant
Al-Hiyasat AS et al. (2023) (19)	Bioceramic	Highest values	Significant
Yusufoglu SI et al. (2019) (20)	Bioceramic	>Resin	Significant
Özyürek EU, Türker SA (2019) (21)	Bioceramic	Time-dependent increase	Significant
Phukan AH et al. (2017) (22)	A H Plus	>ZOE	Significant
Hassan N, Hassan R (2023) (23)	Bioceramic	>resin	Significant
Almohaimede A et al. (2020) (24)	Bioceramic	>Resin	Significant
Ismail SM et al. (2023) (25)	Ca-silicate	>Resin	Not specified
Elnawawy MS et al. (2023) (26)	Bioceramic	> Resin	Significant
Dibaji F et al. (2017) (27)	Bioceramic	>MTA > resin	Significant
Langalia AK et al. (2015) (28)	resin	>control	Significant
Guneser MB et al. (2016) (29)	BioRoot RCS	>control	Significant
Huang G et al. (2023) (30)	Bioactive	>control	Significant
El-Gamal MK et al. (2024) (31)	Warm vertical	Technique effect	Significant
Abdallah Y et al. (2023) (32)	Bioceramic	>resin	Significant
Yendrembam B et al. (2019) (33)	Bioceramic	>resin > MTA	Significant
Issar R et al. (2021) (34)	Resin	Within group difference	Significant
Hajihassani N et al. (2022) (35)	Bioceramic	>Resin	Significant
Muraleedhar AV et al. (2022) (36)	BioRoot RCS	Highest	Significant
Shukri Bm, Gawish As (2024) (37)	Bioceramic	Highest	Significant

Overall, the reported values varied according to material type and experimental conditions. Several investigations demonstrated higher values with calcium silicate–based materials, whereas others reported comparable performance among different sealer categories.

## Discussion

The present systematic review evaluated the influence of different root canal sealers on the mechanical performance of endodontically treated teeth. Based on the qualitative synthesis of 24 studies, obturation generally appeared to improve resistance to root fracture when compared with instrumented but unfilled controls, although the magnitude of this effect varied across materials and study conditions.

Resin-based sealers have traditionally been considered a reference standard because of their favorable physicochemical properties, including dimensional stability and dentinal tubule penetration. Several included studies demonstrated moderate to high reinforcing ability with these materials, consistent with observations reported by Plotino G and Çapar ID.

Calcium silicate–based and bioactive materials have gained increasing attention because of their biomineralization potential and sealing characteristics. Previous investigations have shown that these materials may promote hydroxyapatite deposition and interfacial adaptation with dentin (38). Studies by Guneser MB, Dibaji F, and Huang G reported comparable or higher values with calcium silicate sealers when compared with epoxy resin sealers. However, the reported differences were not consistently statistically significant across investigations.

Zinc oxide eugenol–based sealers generally demonstrated lower reinforcing ability, possibly because of their relatively higher solubility and limited adhesive interaction with dentin.

An important observation in this review was the substantial variation in experimental protocols among studies. Differences in tooth type, canal dimensions, preparation size, irrigation regimens, obturation methods, and loading conditions may all influence mechanical outcomes. Obturation technique also appeared relevant, as cold lateral compaction may generate internal stresses within the root, whereas single-cone approaches rely more heavily on sealer properties.

Due to limited reporting of methodological parameters such as sample size calculation, allocation concealment, and blinding in most included studies, several QUIN domains were scored as “not specified,” resulting in an overall moderate-to-high risk of bias across the included evidence.

Variations in loading direction, crosshead speed, embedding materials, and periodontal ligament simulation further limited direct comparison among studies and prevented quantitative meta-analysis.

### Clinical relevance

Although laboratory testing provides useful information regarding material behavior, direct extrapolation to clinical practice remains limited. Endodontically treated teeth are exposed to cyclic occlusal forces, thermal changes, and restorative procedures that cannot be fully reproduced under experimental conditions (39).

Current evidence suggests that sealer selection alone is unlikely to be the primary determinant of long-term tooth survival. Preservation of remaining tooth structure, ferrule effect, and appropriate restorative rehabilitation remain more critical clinical factors. Therefore, while calcium silicate–based materials show promising laboratory performance, existing evidence does not establish clear clinical superiority over conventional resin-based sealers.

Further standardized *in vitro* studies and well-designed clinical investigations are necessary to clarify the long-term clinical significance of these findings.

### Comparison with previous literature

The current findings are broadly consistent with previously published endodontic literature evaluating the reinforcing effect of root canal sealers on endodontically treated teeth. Earlier narrative reviews by Plotino G and Çapar ID similarly concluded that obturation materials may contribute to improved fracture resistance; however, the reinforcing effect is influenced by multiple variables, including remaining dentin thickness, canal preparation, and obturation technique.

Several *in vitro* investigations included in the present review demonstrated superior or comparable fracture resistance with calcium silicate–based and bioceramic sealers when compared with epoxy resin–based sealers. Studies by Guneser MB, Dibaji F, and Al-Hiyasat AS reported significantly higher fracture resistance values with bioceramic sealers, supporting the growing evidence favoring bioactive obturation materials. Similarly, studies by Elnawawy MS and Hassan N observed improved fracture resistance with calcium silicate–based sealers, particularly when used with single-cone obturation techniques.

Conversely, not all studies demonstrated statistically significant superiority of one sealer category over another. Investigations by Ersoy I and Issar R suggested that obturation technique and methodological variables may exert a greater influence on fracture resistance than sealer composition alone. In addition, several studies reported comparable performance between epoxy resin–based and bioceramic sealers, indicating that the reinforcing effect may be modest and highly dependent on experimental conditions.

The findings of the present review also agree with broader literature emphasizing that fracture resistance is multifactorial and cannot be attributed solely to obturation materials. Factors such as root morphology, extent of structural loss, irrigation protocols, and restorative design have consistently been identified as major determinants of fracture resistance in endodontically treated teeth.

Overall, while a trend toward improved fracture resistance with bioceramic and calcium silicate–based sealers was observed, the available evidence does not conclusively establish the superiority of any single sealer type. The discrepancies among studies are likely related to heterogeneity in experimental protocols, specimen selection, loading conditions, and fracture testing methodologies.

### Strengths of the review

This review followed a structured methodology with predefined eligibility criteria and a comprehensive search across multiple databases, enhancing the breadth of evidence capture. Inclusion of only studies reporting quantitative fracture resistance outcomes allowed focused comparison of mechanical performance. Additionally, the narrative synthesis provides an updated overview of emerging bioactive sealer research.

### Limitations

Several limitations should be acknowledged:

1. Most included studies were *in vitro*, limiting clinical applicability.
2. Significant methodological heterogeneity in tooth selection, obturation techniques, sealer types, and fracture testing protocols prevented quantitative meta-analysis and reduced comparability.
3. Inconsistent outcome reporting and lack of standardized testing methods limited statistical integration.
4. Many studies lacked detailed reporting of randomization and blinding procedures.
5. Potential publication bias cannot be excluded.

### Implications for future research

Future investigations should aim to standardize fracture testing methodologies, including tooth selection criteria, periodontal ligament simulation, and loading conditions. Long-term fatigue testing and aging protocols may better replicate clinical conditions. Most importantly, well-designed clinical trials or prospective cohort studies evaluating survival and fracture incidence are needed to determine whether the reinforcing effects observed in laboratory settings translate into meaningful clinical benefits.

## Conclusion

Within the limitations of the available evidence, root canal sealers appear to provide a supportive reinforcing effect in endodontically treated teeth, with calcium silicate–based and resin-based materials demonstrating generally comparable performance. However, the observed differences were inconsistent across studies and may have been influenced by methodological limitations and potential sources of bias, including inadequate reporting of randomization, blinding, and testing standardization.

In addition, the substantial variability in study protocols prevented sensitivity analysis and quantitative assessment of the influence of bias on the overall conclusions. Structural preservation, ferrule design, and restorative rehabilitation remain more important determinants of long-term tooth survival than sealer selection alone.

Therefore, sealer choice should be based primarily on overall biological, physicochemical, and handling properties until stronger standardized clinical evidence becomes available.

## Data Availability

The original contributions presented in the study are included in the article/Supplementary Material, further inquiries can be directed to the corresponding author.
